# The effect of LED light quality on the carotenoid metabolism and related gene expression in the genus *Brassica*

**DOI:** 10.1186/s12870-023-04326-4

**Published:** 2023-06-21

**Authors:** Katja Frede, Sara Winkelmann, Linda Busse, Susanne Baldermann

**Affiliations:** 1 Leibniz Institute of Vegetable and Ornamental Crops, Plant Quality and Food Security, Theodor-Echtermeyer-Weg 1, 14979 Großbeeren, Germany; 2 University of Bayreuth; Faculty of Life Sciences: Food, Nutrition & Health; Professorship for Food Metabolome, Fritz-Hornschuch-Straße 13, 95326 Kulmbach, Germany

**Keywords:** *Brassica* sprouts, Carotenoids, LEDs, Light quality

## Abstract

**Background:**

New vegetable production systems, such as vertical farming, but also well-established in-door production methods led to the implementation of light emitting diodes (LEDs). LEDs are the most important light sources in modern indoor-production systems and offer the possibility for enhancing growth and specific metabolites *in planta*. Even though the number of studies investigating the effects of LED lighting on vegetable quality has increased, the knowledge about genus variability is limited. In the present study, the effect of different LED spectra on the metabolic and transcriptional level of the carotenoid metabolism in five different *Brassica* sprouts was investigated. Cruciferous vegetables are one of the main food crops worldwide. Pak choi (*Brassica rapa* ssp. *chinensis*), cauliflower (*Brassica oleracea* var. *botrytis*), Chinese cabbage (*Brassica rapa* ssp. *pekinensis*), green kale (*Brassica oleracea* ssp. *sabellica*) and turnip cabbage (*Brassica oleracea* spp. *gongylodes*) sprouts were grown under a combination of blue & white LEDs, red & white LEDs or only white LEDs to elucidate the genus-specific carotenoid metabolism.

**Results:**

Genus-specific changes in plant weight and on the photosynthetic pigment levels as well as transcript levels have been detected. Interestingly, the transcript levels of the three investigated carotenoid biosynthesis genes phytoene synthase (*PSY*), β-cyclase (*βLCY*) and β-carotene hydroxylase (*βOHASE1*) were increased under the combination of blue & white LEDs in the majority of the *Brassica* sprouts. However, only in pak choi, the combination of blue & white LEDs enhanced the carotenoid levels by 14% in comparison to only white LEDs and by ~ 19% in comparison to red & white LEDs.

**Conclusions:**

The effects of light quality differ within a genus which leads to the conclusion that production strategies have to be developed for individual species and cultivars to fully benefit from LED technology.

**Supplementary Information:**

The online version contains supplementary material available at 10.1186/s12870-023-04326-4.

## Background

The consumer demand for high quality vegetables containing health-promoting compounds, but also current and future challenges such as climate change or land and water shortage, led to the development of new vegetable production systems, such as vertical farming. These systems need artificial lighting due to a limited access to natural light. Light emitting diodes (LEDs), which are considered the future of greenhouse lighting [1], offer the advantage of the availability of LEDs emitting specific wavelengths that can optimize the quantum yield of photosynthesis [2], and in addition, can enhance the vegetable quality by influencing the contents of secondary plant compounds determining flavour, colour and health-promoting properties [3]. However, many studies focus on metabolite profiles, whereas the effects and regulatory mechanisms by which specific wavelength affect secondary compounds in various vegetables are not well understood.

**Fig. 1:**
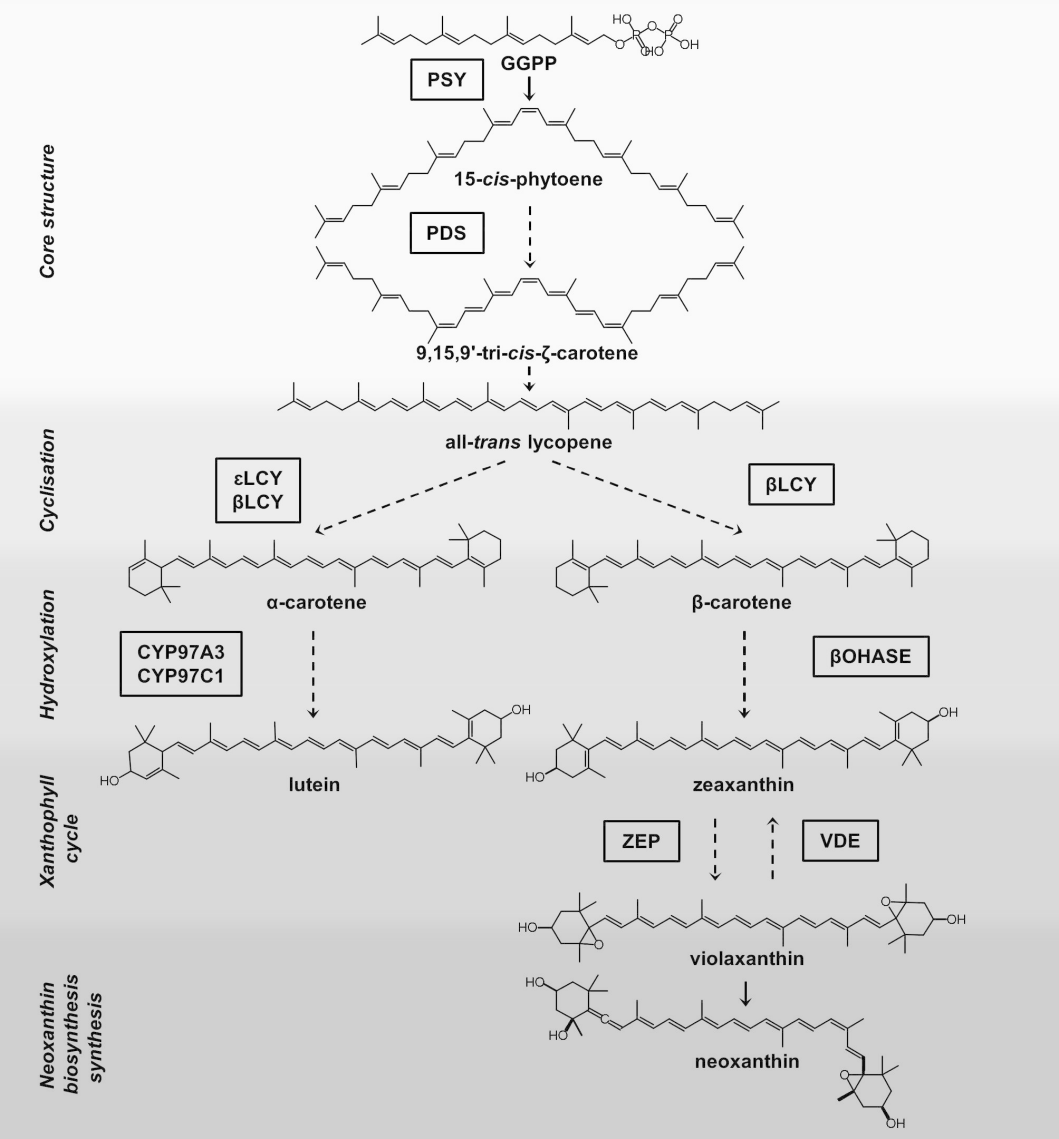
Carotenoid biosynthesis pathway in plants (dashed line: > 1 reaction; continuous line: 1 reaction; GGPP: geranylgeranyl diphosphate; CYP97A3: cytochrome P450 97A3; CYP97C1: cytochrome P450 97C1; βLCY: β-cyclase; εLCY: ε-cyclase; β-OHASE1: β-carotene hydroxylase 1; PDS: phytoene desaturase; PSY: phytoene synthase; VDE: violaxanthin de-epoxidase; ZEP: zeaxanthin epoxidase

Carotenoids are plant pigments belonging to the secondary plant compounds [4], have health-promoting effects in humans [5], and thus, are important for vegetable quality. Carotenoid intake and their concentrations in the body have been correlated with the reduced incidence of chronic diseases, such as type 2 diabetes, cardiovascular diseases and several types of cancer [6]. A major source for carotenoids are green leafy vegetables [6]. The carotenoid biosynthesis is well-known (Fig. 1), however, the regulatory mechanisms determining the contents in vegetables are insufficiently described. In green tissues, carotenoids play a crucial role in photosynthesis [4]. Besides a manipulation of temperature, light regimes during vegetable growth can lead to altered levels [7]. There is only limited knowledge about the underlying mechanisms which specific wavelengths influence the carotenoid biosynthesis pathway in green tissues. Different photo-regulatory mechanisms by photoreceptors [8, 9], the circadian clock [10–12] and an imbalance in the excitation of the photosystems which activates redox control mechanisms [13, 14] are involved in the regulation of the carotenoid metabolism. Light-activated cryptochromes (regulated by blue light) inhibit the COP1-based ubiquitin-ligase and thus rescue the HY5 transcription factor from proteolysis and phytochromes (regulated by red light) inhibit PIF1 transcription factor. Both processes regulate the phytoene synthase (PSY), the first and main rate-determining enzyme in the carotenoid biosynthesis pathway. The excitation of the photosystems by light intensity and quality is regulated through changes of pH-values inside the lumen and the balance of the ratio of reduced and oxidised plastoquinones [15]. This regulates the upper part of the carotenoid biosynthetic pathway and enhances the biosynthesis under blue light as well as the β-carotene hydroxylase (βOHASE) in pak choi [16]. Even though the regulation mechanism are poorly understood the transcripts of the β-cyclase βLCY and the carotenoid cleavage dioxygenase 4 (CCD4) are enhanced by blue light in pak choi too [16].

There have been some studies investigating the effect of specific wavelengths on carotenoids in green, leafy vegetables. In lettuce (*Lactuca sativa* ‘Banchu Red Fire’ or ‘Red Cross’) and sprouting broccoli, blue light increased the carotenoid levels [17–19]. In another study, total carotenoid levels were highest under blue fluorescent lamps in spinach (*Spinacia oleracea* ‘Okame’), but they were enhanced under white and red fluorescent lamps in lettuce (*Lactuca sativa* ‘Redfire’) and under white fluorescent lamps in Komatsuna (*Brassica campestris* ‘Komatsuna’) [20]. On the other hand, slightly higher carotenoid quantities were measured under only white LEDs in comparison to only blue LED or only red LEDs [10], whereas the combination of blue & white LEDs led to significant higher carotenoid levels in pak choi [16].This is interesting since it demonstrates that blue light alone is not sufficient to increase carotenoid levels and shows that other wavelengths of the visual light spectrum in addition to blue light are essential for carotenoid enrichment in pak choi. Thus, the influence of different light qualities on carotenoid concentrations differs between vegetables. Hence, the question arises if wavelength effects are conserved within the same genus. The genus *Brassica* contains economically important crops such as cabbage, cauliflower, broccoli, kale or pak choi leading to a worldwide production of 71 million tonnes *Brassica* vegetables in 2020 [21]. In this study, sprouts of five *Brassica* vegetables were further investigated. Sprouts are harvested and consumed in a stage of expanded cotyledons, are rich in health-beneficial micronutrients and are culinary appreciated because of their taste and colour [22].

In summary, for the production of vegetables rich in carotenoids, the effect of specific wavelength during the growth on carotenoids has to be determined. Therefore, the objective of this study was to quantify the impact of different LED spectra (white or a combination of white & blue or white & red) on the carotenoid metabolism and to clarify if a general recommendation for light regimes of vegetables belonging to the same genus can be made by (1) determining the fresh and dry weight, (2) qualitative and quantitative analysis of the photosynthetic pigments and (3) determining the transcripts of key enzymes of the carotenoid biosynthesis and degradation.

## Materials and methods

### Plant material

Five different sprouts belonging to the genus *Brassica* were investigated: pak choi (*Brassica rapa* ssp. *chinensis* ‘White Celery Mustard’), cauliflower (*Brassica oleracea* var. *botrytis* ‘Neckarperle’), Chinese cabbage (*Brassica rapa* ssp. *pekinensis* ‘Michihili’), green kale (*Brassica oleracea* ssp. *sabellica* ‘Ostfriesische Palme’) and turnip cabbage (*Brassica oleracea* spp. *gongylodes* ‘Delikatess Weißer’) (seed supplier: see Additional file 1). The sprouts were similarly cultivated as previously described in June and July 2020 [10]: Aluminium foil trays (ø ~10 cm) were filled with wet perlite. One gram of seeds per tray (corresponds to 1 replicate) was sown on a wet cellulose cloth which was placed onto the perlite. The seeds were sprayed frequently with water to avoid drying out.

### Growth conditions

**Fig. 2:**
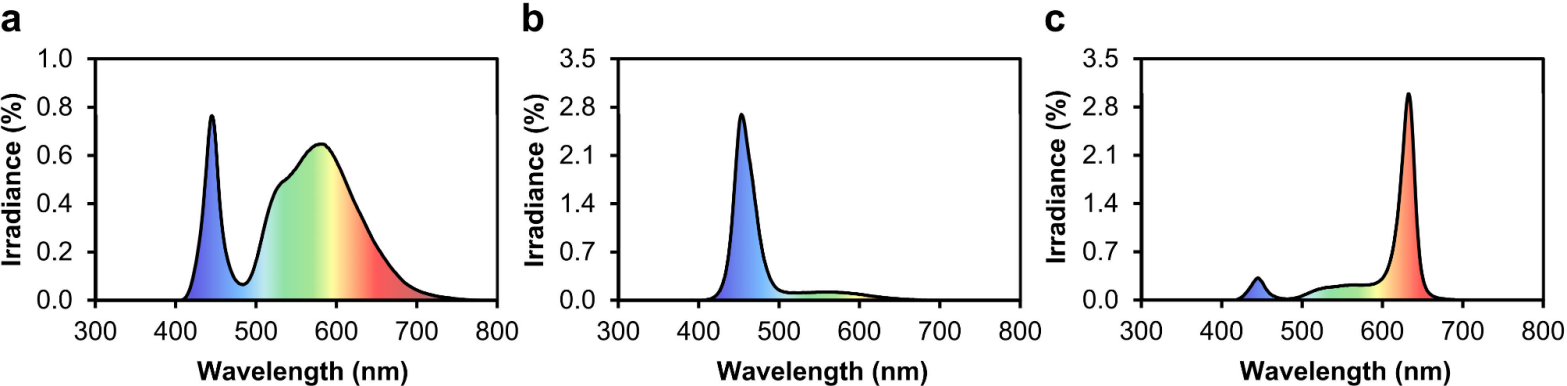
Spectral distribution of **(a)** white, **(b)** blue & white and **(c)** red & white LEDs with filter used in the experiments

The sprouts were grown in darkness for 4 days (25 °C, 75% air humidity). Subsequently, the sprouts were transferred to a growth chamber (25 °C, 75% air humidity) equipped with blue & white (λmax 453 nm), red & white (λ_max_ 633 nm) or only white (λ 404–789 nm) LEDs. The light spectrum of the white LEDs was adjusted with a filter to reduce the amount of blue light and balance the blue and red light [10]. The spectra of the LEDs were analysed with an EPP2000 StellarNet spectrometer (StellarNet, Tampa, FL, USA). The presented LED spectra measured beneath the filter are the means of 3 measuring spots (Fig. 2). The photoperiod in the growth chamber was 12 h (05:00–17:00 h). The photosynthetic active radiation was 90 µmol m^− 2^ s^− 1^ for each light treatment (LI-250 Light Meter, LI-COR, Lincoln, NE, USA). Sprouts were grown for 7 days under the different light spectra and were watered daily. To optimize the uniformity of light treatment, the trays were rotated every day. On day 7 of light treatment, the sprouts were harvested at 09:00 h with 4 biological replicates per LED treatment.

### Sample preparation

In detail, the sprouts of one tray were cut above the perlite surface (cotyledons and hypocotyl) and pooled for 1 replicate. These samples were weighed for fresh mass analysis, frozen immediately in liquid nitrogen and stored at − 80 °C. Two independent experiments (I, II) were performed. For further analysis, the frozen samples were ground to a fine powder under liquid nitrogen. Part of the sample was freeze-dried and carotenoids were analysed using this dried material.

### Dry matter content

The samples were dried in a drying oven at 103 °C until mass constancy. The dry matter content (DMC) was calculated as dry matter per fresh mass in percent.

### Determination of transcripts by RT-qPCR analysis

The primers were designed based on the sequence information available in the Brassica database BRAD (http://brassicadb.org/brad/) (primer pair sequences: see Additional file 1). Total RNA extraction, cDNA synthesis and RT-qPCR was performed as described in Frede et al. [10]. Gene expression was calculated as n-fold induction of the gene of interest in sprouts grown under blue & white or red & white LEDs in comparison to sprouts grown under only white LEDs by the ΔΔCq method using the geometric mean of three reference genes (actin 2, ubiquitin-conjugating enzyme E2 30 and elongation factor 1-alpha) [23, 24].

### Analysis of carotenoids and chlorophylls by LC-ToF-MS

Carotenoids and chlorophylls were extracted and measured as previously described [25]. The separation and detection were performed on a C30 column on an Agilent Technologies 1290 Infinity UHPLC and an Agilent Technologies 6230 TOF LC/MS. External calibration was performed using authentic reference compounds.

### Statistical analyses

SigmaPlot 14.0 (Systat, Erkrath, Germany) was used for statistics. Treatment differences related to LED irradiation by white, white & blue or white & red light were analysed using the one-way ANOVA/Tukey HSD *post hoc* test for each *Brassica* genus. If the assumption of variance homogeneity or normality distribution were violated, the Kruskal–Wallis-test was applied.

## Results

### Fresh mass and dry matter content

**Fig. 3:**
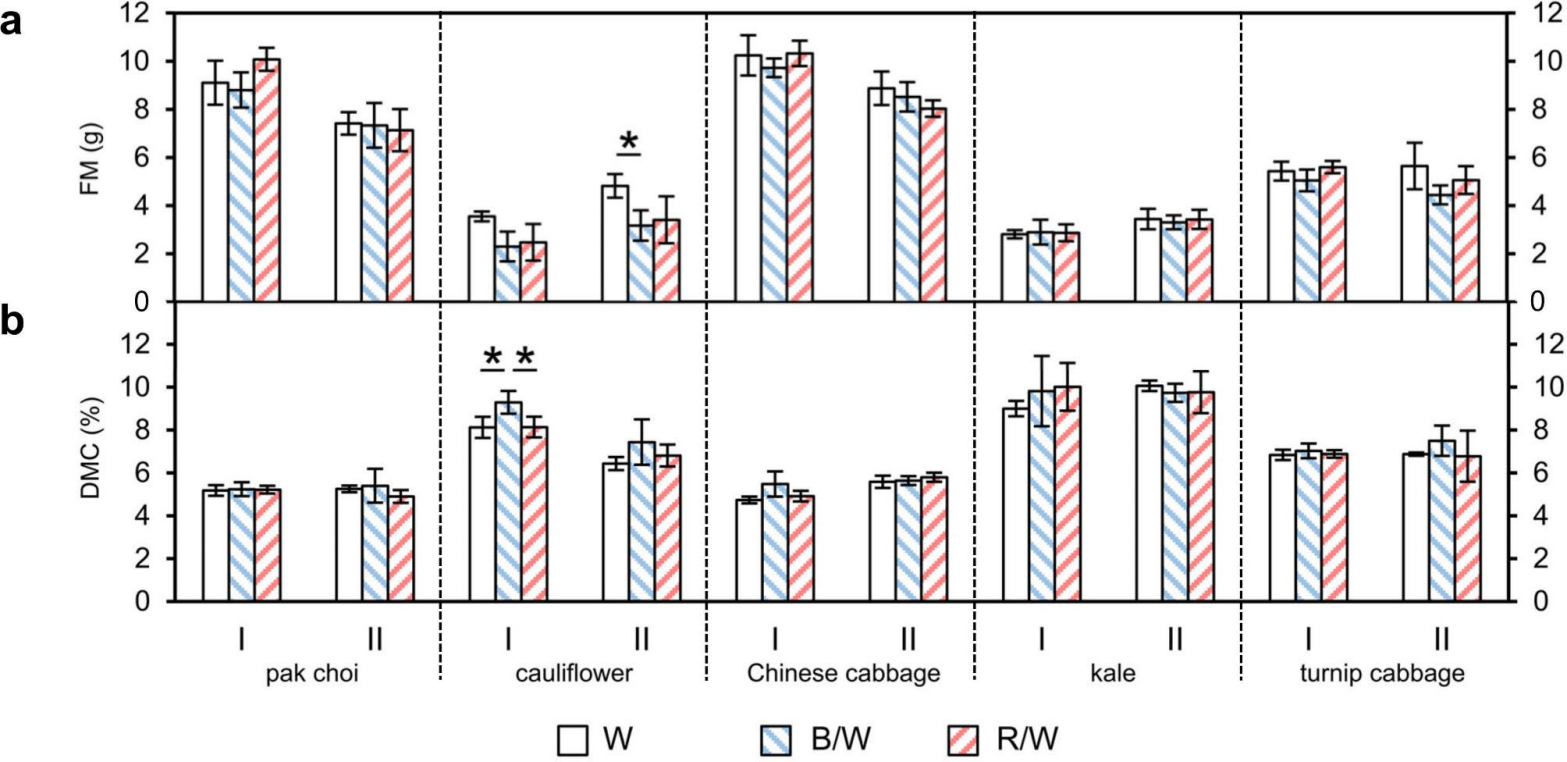
**(a)** Fresh mass (FM) and **(b)** dry matter content (DMC) in different *Brassica* sprouts grown under different light-emitting diode (LED) lamp spectra Sprouts were germinated in darkness for 4 days. Subsequently, the sprouts were grown under white (W) LEDs, blue & white (B/W) LEDs or red & white (R/W) LEDs, and were harvested on day 7 of LED treatment. The DMC was determined as dry matter per fresh mass in percent. Values are presented as mean ± SD (n = 4). Significant differences for each genus are indicated by * (p ≤ 0.05). (I: first experiment; II: repetition experiment)

The fresh mass and the dry matter content were determined in the five different *Brassica* sprouts after growth under the LED treatments. No significant effects of the light quality could be determined in the vast majority of samples that could be confirmed in the repetition experiment (Fig. 3).

### Blue light increases the transcription of carotenoid biosynthesis genes

**Fig. 4:**
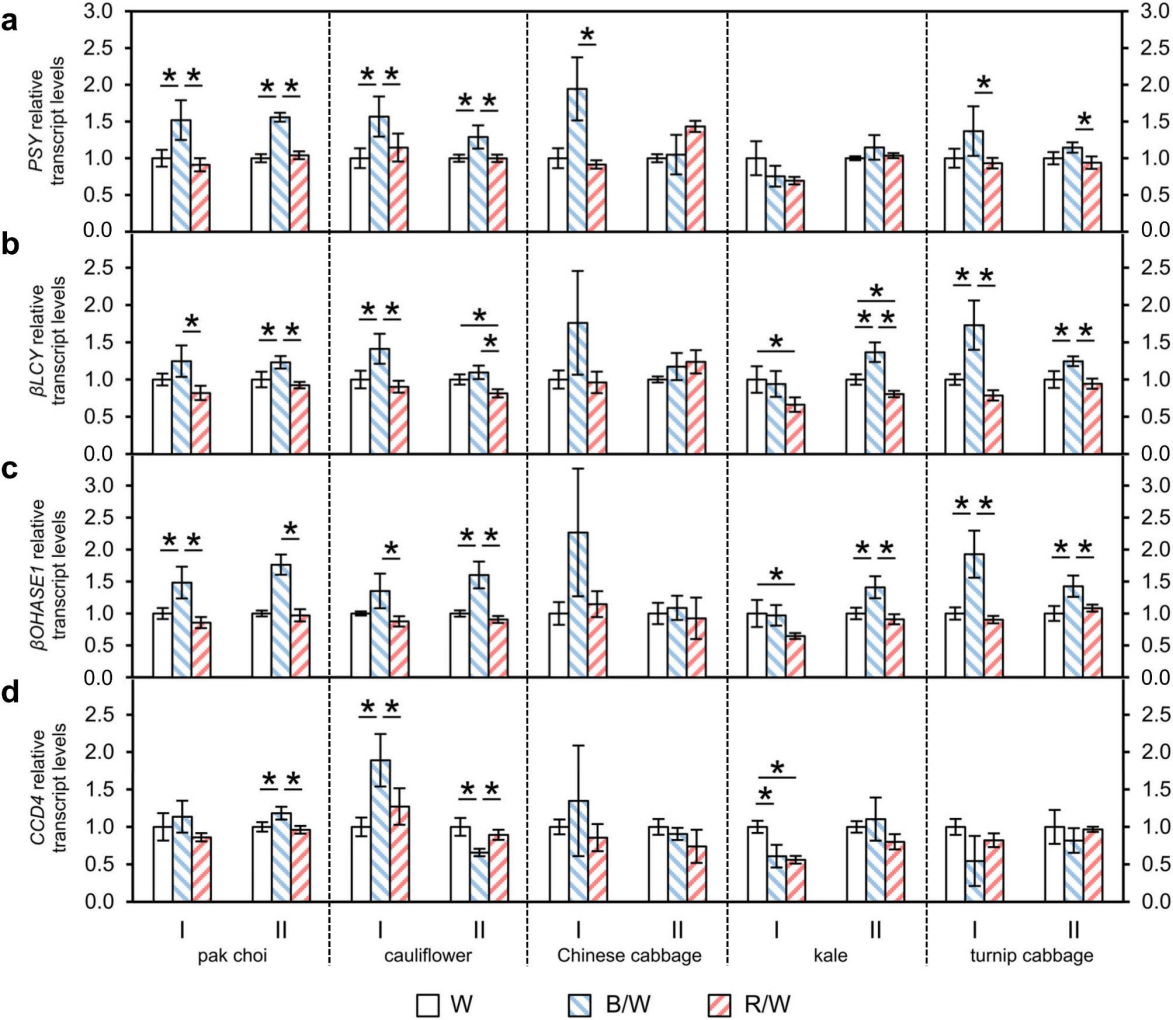
Relative transcript levels of **(a)**  *PSY*, **(b)**  *βLCY*, **(c)**   *βOHASE1* and **(d) **CCD4 grown under different light-emitting diode (LED) lamp spectra. Sprouts were germinated in darkness for 4 days. Subsequently, they were grown under white (W) LEDs, blue & white (B/W) LEDs or red & white (R/W) LEDs. The sprouts were harvested on day 7 of LED treatment. The RNA transcript levels were determined by RT-qPCR analysis. The gene expression was calculated as n-fold of the expression in sprouts grown under only white LEDs by the ΔΔCq method. Values are presented as mean ± SD (n = 3–4). Significant differences for each genus are indicated by * (p ≤ 0.05). (I: first experiment; II: repetition experiment, PSY: phytoene synthase, βLCY: β-cyclase; β-OHASE1: β-carotene hydroxylase 1, CCD4 carotenoid cleavage dioxygenase 4)

In all five *Brassica* spouts, the transcript levels of three carotenoid biosynthesis and one degradation gene under different light qualities were determined as n-fold expression of the expression in sprouts grown under only white LEDs (Fig. 4). The four genes were chosen since they cover different steps of the carotenoid metabolism (see Fig. 1), such as the first biosynthesis step (phytoene synthase), cyclisation (β-cyclase), hydroxylation (β-carotene hydroxylase 1) and degradation (carotenoid cleavage dioxygenase 4), and in addition, these genes were previously shown to react to different light qualities [10]. In the first experiment and confirmed in the repetition experiment, the expression of phytoene synthase (*PSY*) significantly increased in three out of five investigated *Brassica* sprouts after growth under blue & white LEDs in comparison to light spectra with lower blue light percentage, either only white LEDs or red & white LEDs. In pak choi, cauliflower and turnip cabbage, the *PSY* transcript levels were enhanced by ~ 1.2-1.6-fold, whereas in Chinese cabbage the induction of *PSY* under blue & white LEDs was significant only in the first experiment (increased by 1.9 ± 0.4-fold) and there were no significant effects in kale. A similar observation was made for β-cyclase (*βLCY*). Its expression was enhanced by 1.1-1.7-fold by blue & white LEDs in pak choi, cauliflower and turnip cabbage. In kale, blue & white LEDs significantly increased *βLCY* transcript levels only in the repetition experiment (1.4 ± 0.1-fold), while a combination of red & white LEDs significantly reduced the levels to 0.7 ± 0.1 -fold in the first experiment and to 0.8 ± 0.0-fold in the repetition experiment. For β-carotene hydroxylase 1 (*βOHASE1*), blue & white LEDs also increased the expression by 1.4-1.9-fold in pak choi, cauliflower and turnip cabbage. In kale, red & white LEDs significantly reduced the levels to 0.7 ± 0.1-fold in comparison to only white LEDs in the first experiment, while blue & white LEDs led to a significant rise of 1.4 ± 0.2-fold in the repetition experiment. In summary, all three carotenoid biosynthesis genes showed an increase in transcription in the majority of the investigated *Brassica* sprouts when the blue light percentage was increased. However, regarding the degrading enzyme carotenoid cleavage dioxygenase 4 (*CCD4*), no clear results were observed which could be confirmed in the repetition experiment.

### Carotenoid and chlorophyll profiles in ***Brassica*** sprouts after growth under different light qualities

**Fig. 5:**
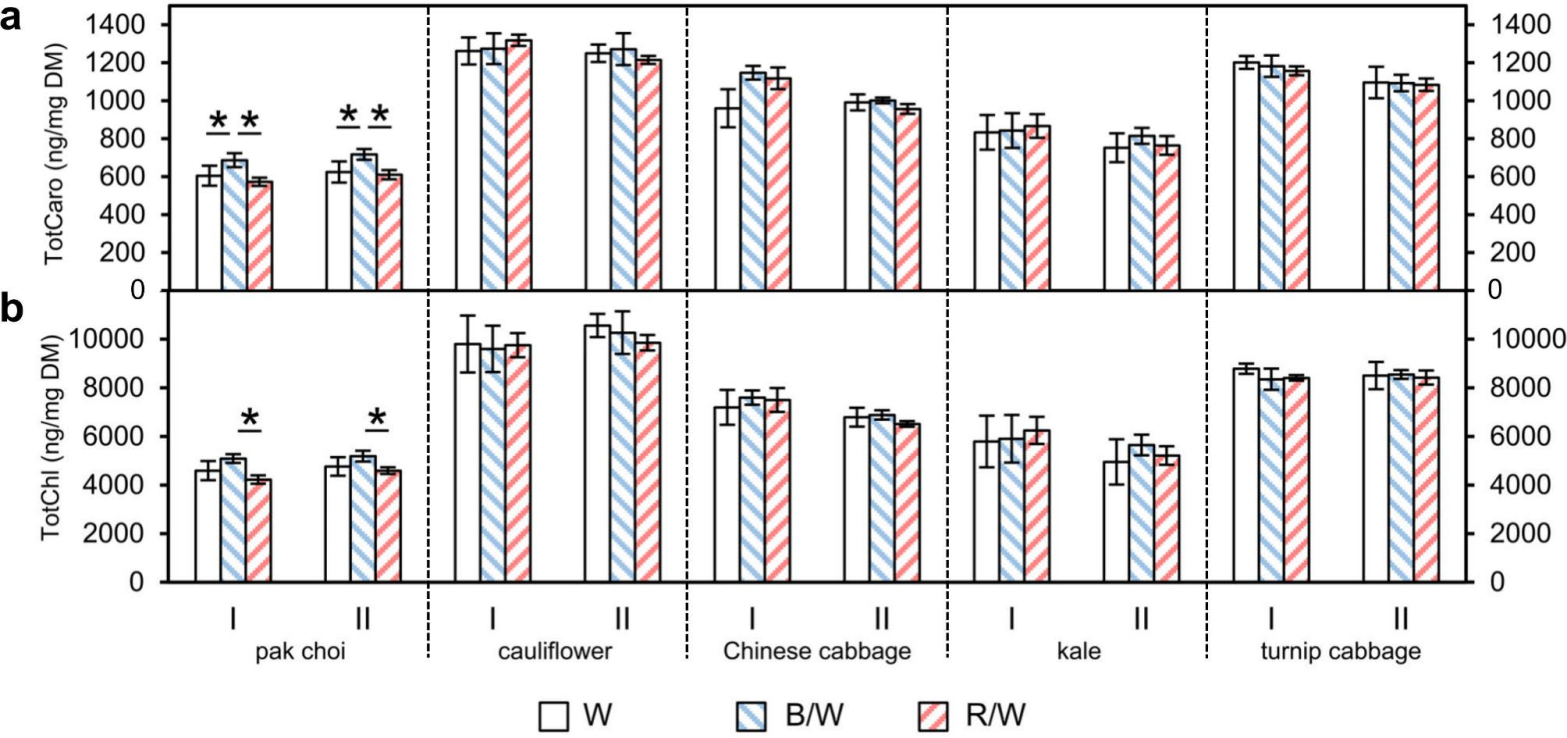
Total **(a)** carotenoid (TotCaro) and **(b)** chlorophyll (TotChl) levels in *Brassica* sprouts grown under different light-emitting diode (LED) lamp spectra. Sprouts were germinated in darkness for 4 days. Subsequently, they were grown under white (W) LEDs, blue & white (B/W) LEDs or red & white (R/W) LEDs. The sprouts were harvested on day 7 of LED treatment. Carotenoids and chlorophylls were measured by LC-ToF-MS analysis. Values are presented as mean ± SD (n = 4). Significant differences for each genus are indicated by * (p ≤ 0.05). (I: first experiment; II: repetition experiment)

The average total carotenoid levels ranged from ~ 600 ng/mg DM in pak choi to ~ 1300 ng/mg DM in cauliflower, while the average total chlorophyll levels ranged from ~ 5000 ng/mg DM in pak choi to ~ 10,000 ng/mg DM in cauliflower (Fig. 5). Only in pak choi a significant effect of the light quality on total carotenoid and chlorophyll levels could be detected. In detail, the combination of blue & white LEDs enhanced the total carotenoid levels by ~ 14% in comparison to only white LEDs and by ~ 19% in comparison to red & white LEDs. In regard to the total chlorophyll levels, the values increased by ~ 10% and ~ 17% under blue & white LEDs in comparison to only white or red & white LEDs, respectively.

**Fig. 6:**
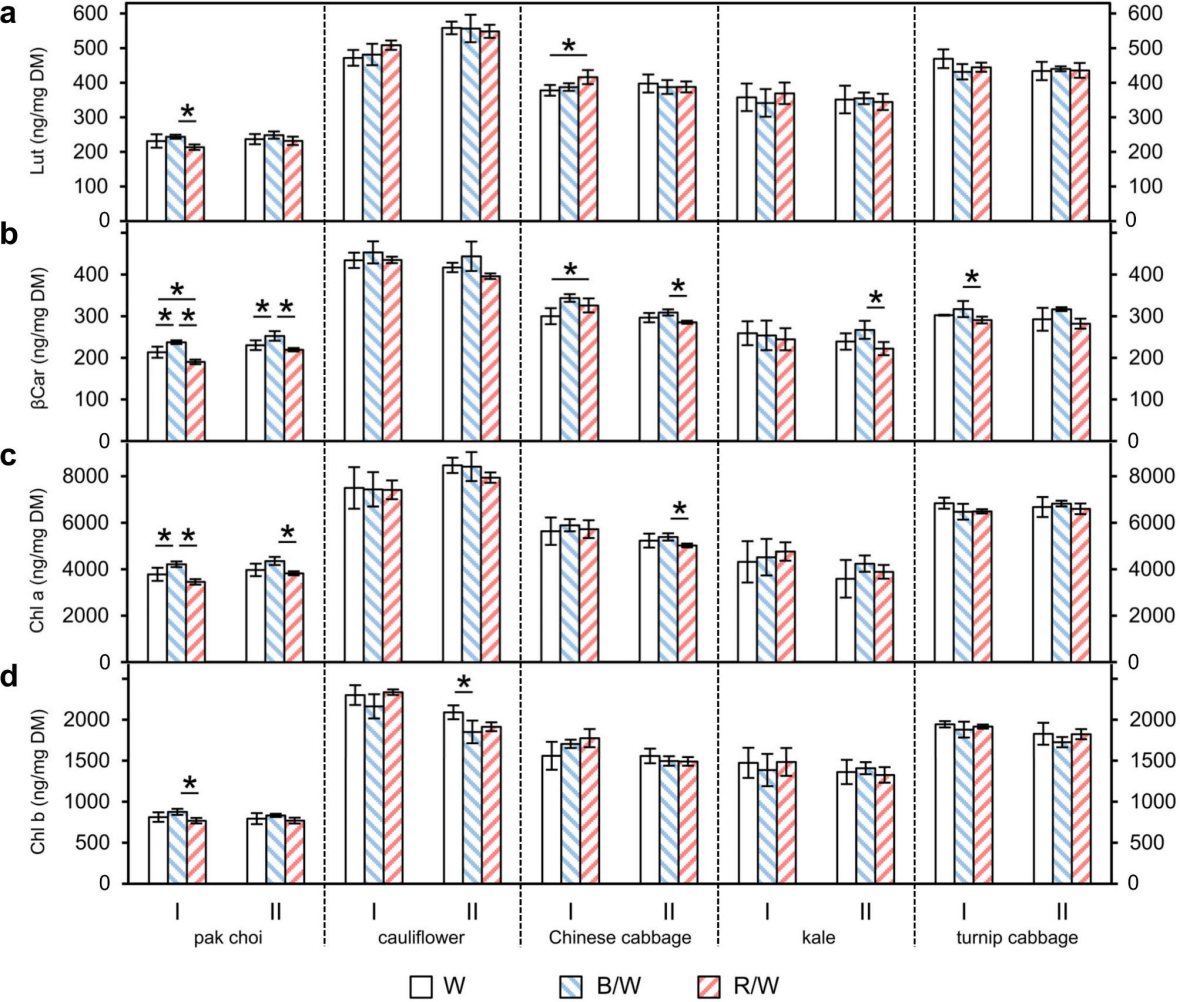
**(a)** Lutein (Lut), **(b)** β-carotene (βCar), **(c)** chlorophyll a (Chl a) and **(d)** chlorophyll b (Chl b) levels in* Brassica* sprouts grown under different light-emitting diode (LED) lamp spectra. Sprouts were germinated in darkness for 4 days. Subsequently, they were grown under white (W) LEDs, blue & white (B/W) LEDs or red & white (R/W) LEDs. The sprouts were harvested on day 7 of LED treatment. Carotenoids and chlorophylls were measured by LC-ToF-MS analysis. Values are presented as mean ± SD (n = 4). Significant differences for each genus are indicated by * (p ≤ 0.05). (I: first experiment; II: repetition experiment)

The individual compounds that could be detected in all *Brassica* sprouts were lutein, β-carotene, neoxanthin and violaxanthin as well as chlorophyll a and chlorophyll b, while α-carotene was found in Chinese cabbage, kale and turnip cabbage (Fig. 6, see Additional file 1). The two dominant carotenoids were lutein and β-carotene, and regarding the chlorophylls, chlorophyll a was the main compound. The dominant carotenoids and the chlorophylls are shown in Fig. 6. Only for pak choi a significant increase in β-carotene and chlorophyll a was shown under blue & white LEDs in comparison to light spetra with lower blue percentage in both experiments.

## Discussion

For the optimization of plant growth and of phytochemical profiles in high quality vegetables, the effects of specific wavelengths on the metabolism have to be investigated more thoroughly. In that regard, the observation was made that the carotenoid metabolism differs in its regulation in photosynthetically active and non-active tissues [26]. Therefore, the results of the present study are discussed by comparing them to studies performed in green tissues.

The fresh mass and the DMC were not influenced by the light quality in the different *Brassica* sprouts or the results were different between the first and second trial for cauliflower. Thus, neither a higher blue nor a higher red percentage of light negatively affected the yield. In other studies, the light quality influenced the fresh and dry mass indicating a species dependent effect. For example, in lettuce (*Lactuca sativa* ‘Banchu Red Fire’, *Lactuca sativa* ‘Red Cross’), red light enhanced the fresh mass in comparison to blue LEDs [17, 18]. In the lettuce (*Lactuca sativa*) cultivar ‘Outredgeous’, additional green LEDs combined with white background LEDs increased the fresh mass 21 days after sowing, while after 28 days after sowing, the combination red plus white LEDs enhanced the fresh mass [27]. Regarding dry mass, Goins et al. [28] presented an increase of shoot dry matter in wheat plants when combining red and blue light in comparison to only red light 40 days after planting. In soybean seedlings, plants exposed to red LEDs showed a smaller plant dry mass in comparison to seedlings exposed to only blue LEDs or to blue combined with red LEDs [29]. Perhaps negative effects of blue light on growth for instance due to reduced cell elongation are not decisive at this developmental stage. Further studies are necessary to elucidate the full potential of LED technology to increase plant yield in respect of developmental stage as well as plant gener aand plant species.

The main goal of the experiments was to investigate how the white light spectrum in comparison to a combination white & blue or white & red light influences the metabolic processes related to carotenoids. The RNA transcripts of four genes were analyzed, three of them coding for carotenoid biosynthetic enzymes and the fourth for a carotenoid catabolic enzyme. For all biosynthesis genes, namely *PSY*, *βLCY* and *βOHASE1*, a higher blue percentage of the light spectrum increased their transcripts in the majority of the sprouts. The transcripts of *CCD4* were not affected by the light quality. CCD4 is an enzyme that is very likely involved in the carotenoid turnover in leaves [30, 31]. However, it was shown in the pak choi cultivar ‘Black Behi’ that the transcription can be influenced by the light quality and shows a diurnal pattern with a peak during the light period [10]. It might be that the point of time for sampling was too early during the light period and that a change could have been determined at a later time point in the investigated species. An enhanced transcription of carotenoid biosynthesis genes was also described in other studies. PSY is a key and rate-limiting step in carotenoid biosynthesis and is related to carotenoid accumulation induced by changes in light regimes. In line with the findings of this study, the number transcripts of PSY were lower under red compared to blue and white light as a single light quality or in combination with white light in pak choi ‘Black Behi’ [10, 13, 16]. The results indicate an involvement of a transcriptional regulation of PSY, since red light regulates phytochrome interacting factors such as PIF1 which acts as transcriptional repressor of PSY, while elongated hypocotyl 5 (*HY5)* acts as transcriptional activator under blue light [7]. In the study of Alrifei et al. [32], enhancing amber and blue light increased the transcription of *PSY* and *εLCY* in *Brassica* microgreens. However, a conclusion about the individual effects of each wavelength cannot be drawn since the light intensities of the two wavelengths were always changed together. In pak choi sprouts of the cultivar ‘Black Behi’, monochromatic blue LEDs induced not only the transcripts of *PSY*, but also of *PDS*, *βLCY*, *εLCY*, cytochrome P450 97A3 (*CYP97A3*), cytochrome P450 97C1 (*CYP97C1*), *βOHASE1*, *ZEP*, *VDE* and *CCD4*, while monochromatic red LEDs decreased them compared to LEDs emitting white light [10]. The cyclisation by βLCY and the hydroxylation by βOHASE1 is not only favoured by monochromatic blue LEDs, but also by its combination with white as shown for pak choi [16] and here in this study for the different *Brassicaceae* sprouts (pak choi, cauliflower and turnip cabbage). Thus, it seems that in accordance to literature blue light increases the carotenoid biosynthetic rate in the genus *Brassica* although this genus is known for its high level of genetic variability [33].

The cultivation under all three different LED treatments led to carotenoid amounts which were similar to levels found in field- or greenhouse-grown vegetables under natural light. In detail, the measured total carotenoid levels ranged from ~ 600 ng/mg DM in pak choi sprouts to ~ 1300 ng/mg DM in cauliflower sprouts. A study conducted in Taiwan with 35 pak choi cultivars presented levels of 440–1110 ng/mg DM during the wet season which was comparable to our growth conditions [34]. In a study conducted with eleven Chinese cabbage cultivars, the total carotenoids levels ranged from 289 to 1001 ng/mg DM [35]. An investigation of 30 kale cultivars found a natural variation of carotenoid amounts of 500–3000 ng/mg DM [33]. These results demonstrated that LED light sources are suitable for carotenoid-rich vegetable production. A further increase could possibly also achieved by special LED light regimes such as sequential light programs [36].

In regard to effects of the light quality on the carotenoid metabolism, only pak choi showed elevated carotenoid amounts under blue & white LEDs which correlated well with transcript levels of the carotenoid biosynthetic genes and the chlorophyll levels. Due to their key role in photosynthesis [4], carotenoids and chlorophylls have to be interrelated which shows in the co-expression of photosynthesis-related and carotenoid biosynthesis genes [12, 14, 37]. Also other studies showed that blue light has the ability to increase carotenoids and other metabolites in leafy vegetables, e.g. in broccoli or kohlrabi [19, 38]. The capability of carotenoids to absorb blue light [39] enables the plant to increase its photosynthesis under blue light, and in addition, protects the plant against this high energy light. In spinach, lettuce, basil and kale, a higher blue light percentage when red and blue LEDs were used together increased the carotenoids more than monochromatic red light [40]. In another study, combining red and blue LEDs or white and blue LEDs instead of using white LEDs alone increased the lutein levels in lettuce (*Lactuca sativa* ‘Outredgeous’) [27]. In spinach (*Spinacia oleracea* ‘Okame’) as well as in further lettuce cultivars (*Lactuca sativa* ‘Red Cross’ and ‘Banchu Red Fire’), blue plus white light or white followed by blue light led to higher carotenoids [17, 18, 20]. However, total carotenoid amounts were enhanced when white plus red light was used for growth of ‘Redfire’ (*Lactuca sativa*), and when ‘Komatsuna’ (*Brassica campestris*) was cultivated under white light [20]. In the other four *Brassicaceae* sprouts namely, cauliflower, Chinese cabbage, kale and turnip, carotenoid levels were the same under the different light regimes indicating different post-transcriptional regulation mechanisms. In line with our study a genus-specific effect in  *Brassica* microgreens was shown for kohlrabi, Mizuna and mustard [22]. Different mechanisms are known in other species such as posttranscriptional regulation of PSY involving phytochrome photoreceptors or changes in the localization of PSY [41]. In order to shed light on posttranscriptional mechanisms on carotenoid accumulation, it is essential to analyze protein levels and enzyme activities in future research.

## Conclusions

Overall, the study demonstrated that *Brassica* species respond to light qualities and the response may vary for individual species and cultivars. The results also demonstrate that *Brassicaceae* sprouts can be produced in controlled environments with comparable quality to outdoor grown vegetables. In addition, the study demonstrated that the transcription of carotenoid biosynthesis genes was increased by the combination of blue & white LEDs in most *Brassica* sprouts, namely in pak choi, cauliflower and turnip cabbage. Surprisingly, this only led to increased carotenoid levels in pak choi. Thus, the responses in carotenoid metabolism to different light qualities are species dependent and measuring the transpiration rate, stomatal conductance and RuBIOSCO activity as well as elucidating the posttranscriptional regulations should help to identify further mechanisms regarding the genus-specific reaction. Hence, no general recommendation for the spectral composition for the *Brassica* genus can be given. The underlying mechanisms defining the carotenoid levels have to be investigated in more detail.

## Electronic supplementary material

Below is the link to the electronic supplementary material.

Supplementary Material 1

## Data Availability

All data generated or analysed during this study are included in this published article [and its supplementary information files].

## Abbreviations

B/W: blue & white LEDs
βCar: β-carotene
CCD4: carotenoid cleavage dioxygenase 4
Chl a: chlorophyll a
Chl b: chlorophyll b
CYP97A3: cytochrome P450 97A3
CYP97C1: cytochrome P450 97C1
DMC: dry matter content
FM: fresh mass
GGPP: geranylgeranyl diphosphate
βLCY: β-cyclase
εLCY: ε-cyclase
LED: light emitting diode
Lut: lutein
βOHASE: β-carotene hydroxylase
PDS: phytoene desaturase
PSY: phytoene synthase
R/W: red & white LEDs
TotCaro: total carotenoids
TotChl: total chlorophylls
VDE: violaxanthin de-epoxidase
ZEP: zeaxanthin epoxidase
W: white LEDs
