# Supplementary material for: The effect of LED light quality on the carotenoid metabolism and related gene expression in the genus Brassica
Source: BMC Plant Biol. 2023 Jun 21;23:328. doi: 10.1186/s12870-023-04326-4 (PMC10283245; doi:10.1186/s12870-023-04326-4)
Supplement: Supplementary file 1 — Supplementary Material 1 [file 12870_2023_4326_MOESM1_ESM.pdf]

Table S1 Seed supplier

Vegetable	Seed supplier
Pak choi (*Brassica rapa* ssp. *chinensis*) ‘White Celery Mustard’	Reinsaat KG, Wolfhoferamt, Austria
Cauliflower (*Brassica oleracea* var. *botrytis*) ‘Neckarperle’	HILD Samen GmbH, Marbach, Germany
Chinese cabbage (*Brassica rapa* ssp. *Pekinensis*) ‘Michihili’	Dürr Samen, Reutlingen, Germany
Green kale (*Brassica oleracea* ssp. *Sabellica*) ‘Ostfriesische Palme’	Sativa Rheinau AG, Rheinau, Switzerland
Turnip cabbage (*Brassica oleracea* spp. *gongylodes*) ‘Delikatess Weißer’	Albert Treppens & Co. Samen GmbH, Berlin, Germany

Table S2 Primers for gene expression analysis by RT-qPCR

**Protein (*gene name*)**	**Primer**
**Carotenoid biosynthesis genes**	
Phytoene synthase (*PSY*)	F: GATACCCTGTCGATATTCAGCCATTTAGAG<br>R: CCCATAACCGGAACGCTCATCA
β-Cyclase (*βLCY*)	F: CCCGTTTGATGTGGATAAGATGGTG<br>R: CCGGCCTAGCGACAAGAGACG
β-Carotene hydroxylase 1 (*β-OHASE1*)	F: TCTTGGCAAATGGAGGGAGGTG<br>R: CAAAGAGAAGCGTGCCAGAGAGC
**Carotenoid degrading gene**	
Carotenoid cleavage dioxygenase 4 (*CCD4*)	F: GCTTTCTTCTGTAACCGCCTCTTC<br>R: CTGTCATGCTCATCGCTAACTTCC
**Reference genes**	
Actin 2 (*ACT2*)[a]	F: ACGTGGACATCAGGAAGGAC<br>R: CTTGGTGCAAGTGCTGTGAT
Ubiquitin-conjugating enzyme E2 30 (*UBC30*)	F: CACTGGCAAGCAACTATTATGGGTCC<br>R: CATTGCTGTTGATGTTCGGGTGATA
Elongation factor 1-alpha (*EF1α*)	F: CCCAAGTTTTTGAAGAATGGTGA<br>R: ACGGTCTGCCTCATGTCCCT

[a] Wiesner M, Zrenner R, Krumbein A, Glatt H, & Schreiner M. Genotypic variation of the glucosinolate profile in pak choi (*Brassica rapa* ssp. *chinensis*). J Agric Food Chem. 2013;61(8):1943-53. https://doi.org/10.1021/jf303970k

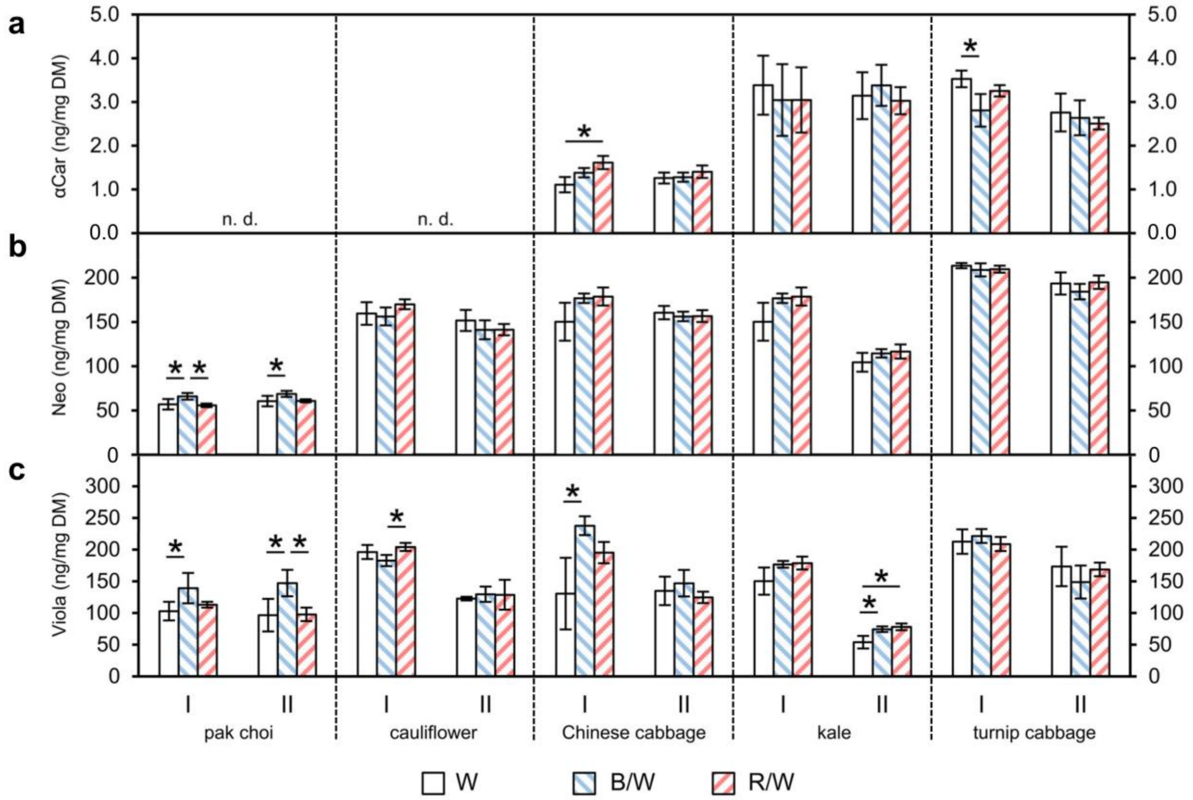

Fig. S1 (a) α-Carotene (αCar), (b) neoxanthin (Neo) and (c) violaxanthin (Viola) levels in *Brassica* sprouts grown under three different light qualities. Sprouts were germinated in darkness for 4 days. Subsequently, they were grown under white (W) LEDs, blue & white (B/W) LEDs or red & white (R/W) LEDs. The sprouts were harvested on day 7 of the LED treatment. The carotenoids were determined by LC-ToF-MS analysis. Values are presented as mean ± SD (n = 4). Significant differences for each genus are indicated by * ($p \leq 0.05$). (I: first experiment; II: repetition experiment; n. d.: not detectable)
